# Detection of *Mycobacterium avium* subsp. *paratuberculosis* in Australian Cattle and Sheep by Analysing Volatile Organic Compounds in Faeces

**DOI:** 10.3390/s24237443

**Published:** 2024-11-21

**Authors:** Rachel Hodgeman, Christian Krill, Simone Rochfort, Brendan Rodoni

**Affiliations:** 1 Agriculture Victoria, AgriBio, La Trobe University, Bundoora, VIC 3086, Australia; christian.krill@deeca.vic.gov.au (C.K.); simone.rochfort@agriculture.vic.gov.au (S.R.); brendan.rodoni@agriculture.vic.gov.au (B.R.); 2 School of Applied Systems Biology, AgriBio, La Trobe University, Bundoora, VIC 3086, Australia

**Keywords:** *Mycobacterium avium* subsp. *paratuberculosis*, Johne’s disease, gas chromatography–mass spectrometry with solid-phase micro-extraction (SPME-GC-MS), electronic nose (eNose), volatile organic compounds

## Abstract

Paratuberculosis is a debilitating disease of ruminants that causes significant economic loss in both cattle and sheep. Early detection of the disease is crucial to controlling the disease; however, current diagnostic tests lack sensitivity. This study evaluated the potential for volatile organic compounds (VOCs) detected by gas chromatography and an electronic nose (eNose) for use as diagnostic tools to differentiate between Map-infected and non-infected cattle and sheep. Solid-phase micro-extraction gas chromatography–mass spectrometry (SPME GC-MS) was used to quantify VOCs from the headspace of faecal samples (cattle and sheep), and partial least squares–discriminant analysis (PLS-DA) was used to determine the suitability as a diagnostic tool. Both the cattle and sheep models had high specificity and sensitivity, 98.1% and 92.3%, respectively, in cattle, and both were 100% in sheep. The eNose was also able to discriminate between Map-infected and non-infected sheep and cattle with 88.9% specificity and 100% sensitivity in sheep and 100% specificity and sensitivity in cattle. This is the first time that VOC analysis by eNose and GCMS has been used for identification of Map in cattle and sheep faeces. GCMS also allowed the identification of putative disease biomarkers, and the eNose diagnostic capability suggests it is a promising tool for point-of-care diagnosis for Map detection on farms.

## 1. Introduction

*Mycobacterium avium* subsp. *paratuberculosis* (Map) is the causative agent of Johne’s disease (JD), which is a debilitating disease-causing chronic enteritis in domestic and wild ruminants. The disease is spread worldwide, causing significant economic loss to the dairy industry due to reduced milk production, culling of animals, and reduced slaughter value [1]. There are no effective treatments for Johne’s disease and control is complicated due to the progression and presentation of the disease. Infected animals slowly advance through four stages of the disease. In stage one (silent infection), there are no observable or detectable effects of the disease. There are also generally no observable effects in stage two, but some animals may start shedding low numbers of bacteria intermittently, and there is some evidence of cellular and humoral response. Stages three and four are characterised by the onset and progression of clinical disease [2], which can start from 2 years of age and up to 10 years after infection [3].

Detection and control of JD is particularly challenging during the first few years of life during stage one of the disease when animals are not shedding the bacteria. Current diagnostic tests such as culture, ELISA, and PCR have limitations for detecting the disease during stages one and two and lack sensitivity due to low or non-existent faecal shedding and antibody titres [4]. Currently, the most sensitive method for detection of Map is culture directly from faeces; however, due to the long replication time of the bacteria, this method can take up to three months for a definitive result [5,6]. Test and cull programmes for control of JD on farms are the most widely deployed control measures, but they are largely ineffective as they cannot control the spread of disease due to the poor sensitivities of the current testing methods. For successful control of disease, it is crucial to have a diagnostic tool that can detect early infection.

Bacteria emit volatile organic compounds (VOCs), which can be identified and quantified in biological samples such as fluids or faeces [7]. At low temperatures, these compounds transform into a gaseous state that is measurable by mass spectrometry (MS), or gas chromatography–mass spectrometry (GC-MS) [8]. There have been significant advancements in recent years in the use of VOC analysis for the detection of human and animal diseases directly from biological samples. Breath analysis has successfully been used for the diagnosis of tuberculosis in human patients [9,10] and for detection of the *Mycobacterium tuberculosis* complex in wild boars [11]. Faecal pathogens that have been successfully detected in the headspace of cultures and faeces, including *Clostridium difficile* and *Campylobacter jejuni*, as well as rotavirus, enteric virus, and *Vibrio cholera* 01 (9). VOCs associated with diseases such as Crohn’s disease, ulcerative colitis, and irritable bowel syndrome have also been detected in the headspace of faeces [12]. VOCs have been used to detect *E. coli* [13], *Aspergillus*, and *Fusarium spp.* [14] from pure cultures.

Sensor arrays such as in an electronic nose (eNose) are designed to mimic the olfactory system of the human nose to detect VOCs [15]. eNose sensors have been used to differentiate between respiratory pathogens [16] and *Mycobacterium tuberculosis* [17,18] and have been used on the headspace of serum to identify infected and non-infected cattle and badgers with *Mycobacterium bovis* [19].

In this study, SPME-GC-MS has been used to identify VOCs in the headspace of faecal cultures and directly from faeces from both cattle and sheep. These VOCs were used in PLS-DA models to determine if they could be used to differentiate between Map-positive and Map-negative samples. An electronic nose was also evaluated to determine if this technology could be used as a point of care tool to detect Map in faeces from both cattle and sheep.

## 2. Materials and Methods

### 2.1. Panel of Samples, Culture and PCR Preparation

Faecal samples were obtained from cattle and sheep faeces originating from different animals and herds/flocks from Victoria, Australia, with approval from the Animal Ethics Committee in Victoria (permit number 2022-05). The presence or absence of Map was initially determined at the time of the study by cultural isolation [20] and a Map-specific qPCR as previously described (Hodgeman et al. [21]. The strain type of all the isolates was also determined by the IS1311 PCR and REA as previously described [20]. After initial processing, if the samples could not be tested immediately, they were stored at −80 °C until preparation for this study. See Supplementary Table S1 for a complete description of the faecal samples used in this study.

### 2.2. Sample Preparation

#### 2.2.1. Cultures

For initial method development, 12 faecal samples that consisted of 9 cattle (8 confirmed as culture-positive and 1 confirmed as culture-negative) and 3 sheep (2 confirmed as culture-positive and 1 confirmed as culture-negative) were prepared for culture as previously described [20], with the exception they were cultured in 20 mL headspace vials. Each faecal sample was cultured in triplicate, and Map cultures were prepared on different days to be analysed by SPME-GC-MS after 2, 4, 6, 8, 10, and 12 weeks post inoculation. For each culture and time point, Map status was confirmed by the IS900 PCR as previously described [20].

#### 2.2.2. Faeces

For direct faecal analysis, 48 Map-negative faeces and 28 Map-positive cattle faeces and 30 Map-negative and 4 Map-positive sheep faeces (Supplementary File S1: Table S1) were prepared in triplicate for direct SPME-GC-MS analysis by placing 3 g of faeces directly into 20 mL clear headspace vials with magnetic screw top lids and polytetrafluorethylene (PTFE) septa. Only faecal samples that had tested positive by both culture and PCR or negative by culture and PCR were used as part of the validation panel for VOC analysis to ensure there were no discrepancies between results. Peak areas from these data were exported to Excel and used as input to Matlab (ver 2023b, Mathworks) [22] to produce PLS-DA score plots to determine if this method could be used as a diagnostic test.

### 2.3. VOC Analysis

Solid-phase micro-extraction (SPME) was used to sample the headspace of both the cultures and faeces. A carbon WR/PDMS SPME Arrow (Gerstel GmbH & Co., KG, Mulheim, Germany), 1.1 mm diameter, 20 mm phase length, and 120 µm phase thickness, was used for all measurements. Before SPME Arrows were used for the first time, they were conditioned at 270 °C for 30 min (according to the manufacturer’s instructions). Every day before measurements were taken, a blank run of the arrow was performed to ensure the SPME coating was clean and there was no uncontrolled bleeding. Map cultures and faeces were conditioned at 37 °C for 5 min, and extraction of volatiles was performed for 2 min at 37 °C. The Arrow was reconditioned at 270 °C for 5 min before each sample and for 2 min directly after injection. SPME fibres were thermally desorbed in the GC-injector for 60 s at an injector temperature of 270 °C and a 40:1 split. The inlet liner was a 2 mm ID straight HS-type liner (Agilent, Mulgrave, Australia). The column was an Agilent DB-624 30 m length, 0.25 internal diameter, and 1.4 µm film thickness. The carrier gas was helium at a constant flow rate of 1.2 mL/min. The temperature programme was set at 40 °C for 5 min, with a ramp of 3 °C/min to 100 °C, followed by 10 °C/min to 150 °C, with a ramp of 100 °C/min to 240 °C held for 5 min. The detector used was an Agilent 7250 Q-TOF operated in MS1 mode, acquiring a mass range of 45–350 amu at 5 spectra/s in centroid. The EI source was held at 200 °C, and emission was fixed at 1µ Amp and 70 eV.

### 2.4. Data Analysis

Volatiles were characterised by interrogating raw data in Mass Hunter Qualitative v.10.0. by their *m*/*z* and retention time and labelled according to elution time (UK1-UK76). Mass Hunter Quantitative v.10.0 was used for relative quantification of these VOCs. Peak areas were exported to Excel and used as input to PLS Toolbox (ver 9.2.1 Eignevector) Matlab (ver 2023b, Mathworks) [22] for principal component analysis (PCA) and partial least squares–discriminant analysis (PLS-DA). Compound intensities were pre-processed using autoscale or mean centering prior to analysis. For PLS-DA, the data were split into test and validation using the Kennard–Stone approach, with 75% of the data retained for development of the model. The PLS-DA model used cross validation with venetian blinds (with 10 splits and a blind thickness of 1).

To determine the specificity and sensitivity of the test based on PLS-DA predictive modelling, the following calculations were used [23]:

$$\mathrm{Sp}=\frac{TN}{TN+FP}\;\mathrm{Se}=\frac{TP}{TP+FN}$$

where *TN* = true negative, *FP* = false positive, *TP* = true positive, and *FN* = false negative.

Based on these calculations, the predicted sensitivity of the test is 97.8% and the predicted specificity is 90.5%.

The class error was calculated as follows:

Class Err. = average of false-positive rate and false-negative rate for class, = 1 − (sensitivity + specificity)/2.

### 2.5. Identification of Putative Biomarkers

Putative biomarkers were identified by matching the spectra of chromatographic peaks against the NIST library (NIST 2205 Gatesburg, PA, USA) and, where possible, verified by comparison to GC retention times and mass spectra of reference standards. Thirteen such standards (pentanal, hexanal, 1-octen-3-ol, 2-octanone, ethanthiol, 3-carene, p-cymene, phenol, acetone, 2-butanol, D-limonene, 1-butanol 3-methyl, and 1-butanol 2 methyl from Sigma Aldrich, St. Louis, MO, USA) were prepared by diluting approximately 50 mg of each reference standard in 20 mL and 10 mL of isopropanol (Sigma Aldrich, Molecular grade)supplier and grade) and 1 µL of these dilutions were added to a headspace vial. The headspace was measured as previously described.

### 2.6. Validation of GC-MS-Based Models for Faeces

#### 2.6.1. eNose Data Collection and Model Training

**Figure 1:**
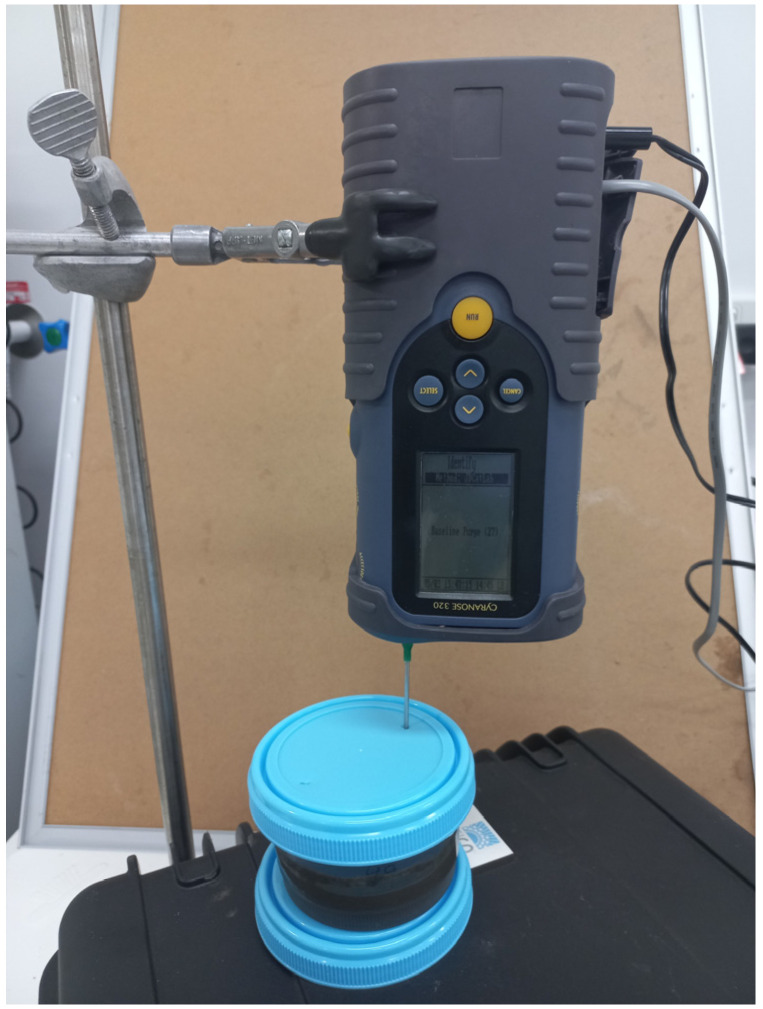
Illustration of the Cyranose^®^ 320 eNose^®^ apparatus for sampling cattle and sheep faeces in 200 mL plastic containers.

The Cyranose^®^ 320 eNose^®^ was trained to distinguish between Map-positive faeces and Map-negative faeces in both cattle and sheep. The Cyranose^®^ 320 eNose^®^ (Sensigent, Baldwin Park, CA, USA) is a portable olfactory system that is a combination of a gas sampling unit and a sensory array and consists of 32 different thick film metal oxide sensors. The Cyranose^®^ 320 eNose^®^ has two pumps, one for pulling samples through the sensor array and one for transferring filtered air into the sensor array. The filtered air is also used as a baseline and to remove residual volatile compounds, and the sensor response from the sample gas is measured in comparison to the filtered air. For training the eNose in the laboratory, 100 g of confirmed Map-positive or Map-negative faeces was weighed into a 200 mL plastic container and incubated at 37 °C for one hour. Two 3 mm diameter holes were punched into the lid of the container at opposite ends, and the eNose snout was inserted to approximately 15 mm above the surface of the faeces through one of the holes (Figure 1). For establishing the internal prediction model, the eNose was presented with 10 individual faeces for each class (infected and non-infected faeces) using the ‘Training’ mode as described by the manufacturer [24] (Table 1). The Cyranose^®^ 320 eNose^®^ was also trained in the field to distinguish between Map-positive faeces and Map-negative faeces using the same method as above, except the faeces were not incubated at 37 °C for one hour. The faeces were taken directly from the animal into large 500 mL collection pots, and approximately 100 mL was decanted into 200 mL pots and incubated at air temperature for 10–15 min to establish the headspace before analysis. Once training of the eNose was completed, a preliminary evaluation of the model was performed by performing an internal cross-validation of the infected and non-infected classes to determine the discrimination ability of the eNose. The eNose software employing a Canonical Discriminant Analysis (CDA) (a special case of linear discriminant analysis, also a supervised learning classifier) was used to analyse the data as it allowed clear separation of the infected and non-infected classes.

A limitation of the eNose software was that only 10 positive and 10 negative samples could be used to develop the training model. To allow comparison to the GCMS method, the sensor data (initial 20 training samples and subsequent test samples) was also exported and analysed using PLSToolbox to create PLS-DA models. Pre-processing was either mean centering or autoscaling, with the data split into a training (75%) and test set (25%) using the Kennard–Stone algorithm. Premutation testing was performed (*n* = 50) to test model robustness.

#### 2.6.2. Validation of eNose

For the validation of the eNose for cattle, the original 20 samples used for training were included with an additional 22 Map-negative and 12 Type-C Map-positive cattle faeces. For sheep, the original 10 samples used for training were included with an additional 13 negative and 8 positive sheep faeces (Supplementary File S1: Table S1). To validate the eNose capability for identifying samples, the eNose is used in ‘Identify’ mode instead of the ‘Training’ mode. The sensor data were exported to Excel and analysed using Matlab. Data were analysed using autoscaling or mean centrering with data split using Kennard–Stone algorithm with Euclidean distance to retain 75% of the samples for training.

## 3. Results

### 3.1. Culture and qPCR

Ninety five cattle and sixty sheep faeces samples were tested by culture and qPCR to determine the presence/absence of Map prior to VOC analysis. Of the 95 cattle faeces samples, 36 were cultured and PCR-positive and 59 tested negative for Map, and of the 60 sheep, faeces 14 were cultured and PCR-positive and 46 were Map-negative (Supplementary Table S1). The 12 cultures that were used for VOC analysis at 2, 4, 6, 8, 10, and 12 weeks post inoculation were also subjected to PCR at the same time points. Two of the Map-positive cattle cultures were detected by PCR at week 8, a further culture at week 10, and all cultures were positive at week 12. All Map-positive sheep cultures were detected by PCR at 12 weeks post-inoculation (Supplementary Table S2).

### 3.2. Assessment of Volatile Organic Compounds from Cultures

**Figure 2:**
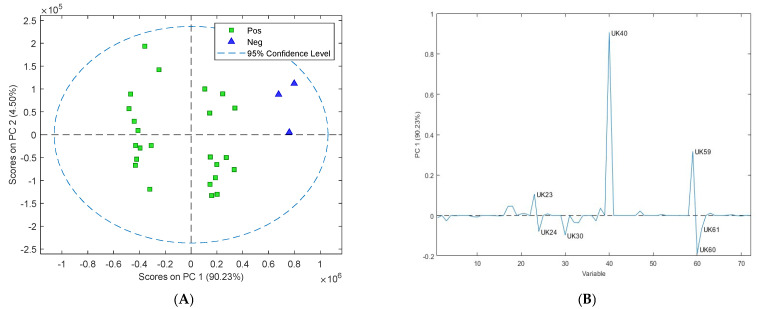
(**A**) Two-dimensional principal component analysis (PCA) scores plot of the volatile organic compounds collected from the headspace of Map-positive (green square) and Map-negative (blue triangle) cattle cultures at four weeks of culture showing grouping pattern of samples according to the first two principal components. (**B**) The contribution of individual volatiles to principal component 1 is shown in the loadings plot for the cattle cultures at four weeks of culture, with seven putative biomarkers influencing separation on PC1 (UK23, UK24, UK30, UK40, UK59, UK60, and UK61).

VOC analysis of Map cultures resulted in the identification of 72 compounds in the headspace of the sample vials. Evaluation of the PCA plot of the cattle culture data from week 2 to week 12 showed that Map-infected faecal cultures could be differentiated from non-infected faecal cultures at 4 weeks of culture (Figure 2A) with clear separation of the positive and negative samples on PC1. The loading plot (Figure 2B) revealed that there were seven main VOCs responsible for this separation. They were identified as UK23—pentanal, UK24—3-pentanone, UK30—1-butanol 3-methyl, UK40—hexanal, UK59—1-octen-3-ol, UK60—6-octen-2-one (Z), and UK61—2-octanone (Table 2). UK23, 40, and 59 were upregulated in the negative samples, and UK24, 30, 60, and 61 were all upregulated in the Map-positive samples. 

**Figure 3:**
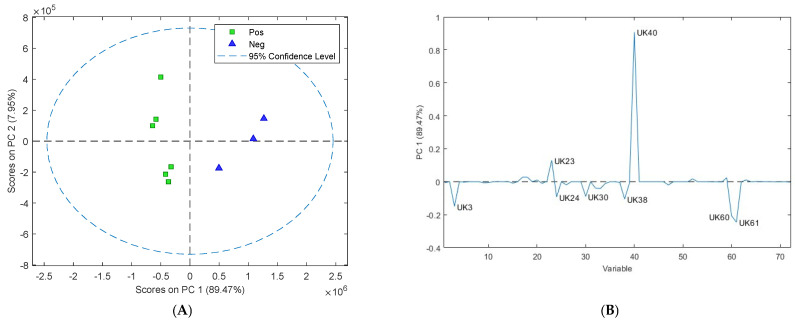
(**A**) Two-dimensional principal component analysis (PCA) scores plot of the volatile organic compounds collected from the headspace of Map-positive (green square) and Map-negative (blue triangle) sheep faecal cultures at six weeks of culture showing the clustering pattern of samples according to the first two principal components. (**B**) The contribution of individual volatiles to principal component 1 is shown in the loadings plot for sheep cultures at six weeks of culture, showing eight putative biomarkers responsible for separation on PC1 (UK3, UK23, UK24, UK30, UK38, UK40, UK60, and UK61).

Evaluation of the PCA scores plot (Figure 3A) of the sheep cultures from week 2 to week 12 showed that Map-infected faecal cultures could be differentiated from non-infected faecal cultures at 6 weeks of culture (Figure 3A). The loadings plot (Figure 3B) revealed eight VOCs had a significant effect on this separation (UK3—dimethylamine, UK23—pentanal, UK24—3-pentanone, UK30—1-butanol 3-methyl, UK38—1-butanol 2-methyl, UK40—hexanal, UK60—6-octen-2-one (Z), and UK61—2-octanone) in the sheep cultures (Table 2). UK3, 24, 30, 38, 60, and 61 were upregulated in the positive sheep cultures, and UK23 and 40 were upregulated in the negative cultures. Six of the compounds (pentanal, 3-pentanone, 1-butanol, 3-methyl, hexanal, 6-octen-2-one (Z), and 2-octanone) were present in both the cattle and sheep Map-positive cultures.

### 3.3. Assessment of Volatile Organic Compounds from Cattle Faeces

**Figure 4:**
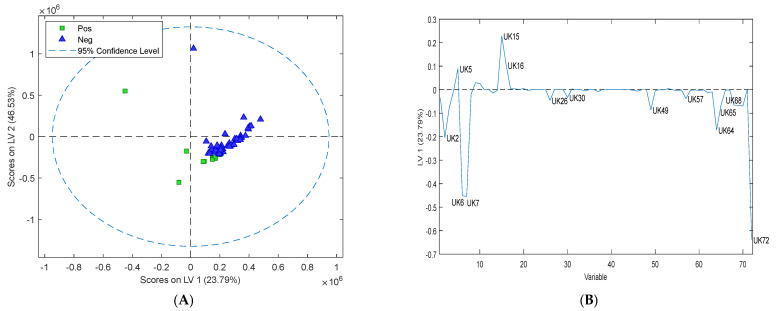
(**A**) The PLS-DA plot of the volatile organic compounds collected from the headspace of cattle faeces showing grouping pattern of Map-positive (green square) and Map-negative (blue triangle) cattle faeces according to the first two latent variables (LV). (**B**) The contribution of individual volatiles to LV1 and LV2 of cattle faeces showing ten putative biomarkers important for separation on LV1 (UK2, UK5, UK6, UK7, UK15, UK16, UK49, UK64, UK65, and UK72).

VOC emissions from faecal samples were analysed by comparing measurements of Map-negative cattle faeces and Map-positive cattle faeces. The Map status of faeces was first determined by both culture and qPCR (Supplementary Table S1). The PLS-DA plot of cattle faeces (Figure 4A) indicated that Map-positive samples could be differentiated from Map-negative samples. The classification errors for the calibration, cross validation, and prediction (i.e., 25% of the data were withheld from the original model) were 6.3%, 8.3%, and 4.8%, respectively. The predicted sensitivity of this test model is 92.3%, and the predicted specificity is 98.1%. Permutation testing (*n* = 50) returned *p*-values less than 0.01, suggesting that the model was not overfitted.

Out of the 72 detectable VOCs, the loading plot (Figure 4B) revealed 10 compounds that could differentiate between Map-positive and Map-negative cattle faeces (Table 2). The 10 putative biomarkers were UK2 (methanthiol), UK5 (acetone), UK6 (dimethyl sulfide), UK7 (ethanthiol), UK15 (2-butanone), UK16 (2-butanol), UK49 (3-carene), UK64 (p-cymene), UK65 (D-limonene), and UK72 (phenol 3-methyl). Seven putative biomarkers, UK2, 6, 7, 49, 64, 65, and 72, were upregulated in the Map-positive cattle faecal samples, and three putative biomarkers, UK5, 15, and 16, were upregulated in the negative cattle faecal samples.

### 3.4. Assessment of Volatile Organic Compounds in Sheep Faeces

**Figure 5:**
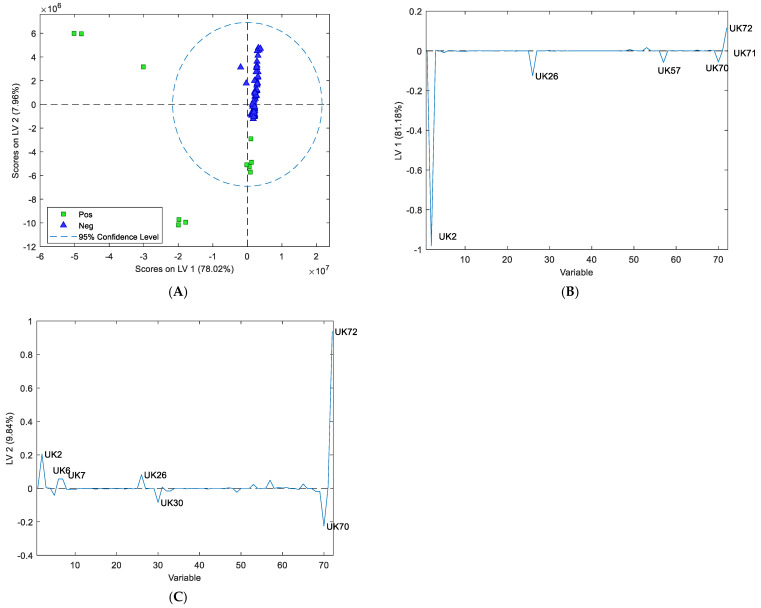
(**A**) The PLS-DA plot of the volatile organic compounds collected from the headspace of sheep faeces showing grouping pattern of Map-positive (green square) and Map-negative (blue triangle) sheep faeces according to the first two latent variables. (**B**,**C**) The contribution of individual volatiles to LV1 and LV2 of sheep faeces showing eight putative biomarkers important for separation on LV1 (UK2, UK6, UK7, UK26, UK30, UK57, UK70, and UK72).

VOC emissions for sheep faecal samples were determined by comparing measurements of Map-negative sheep faeces and Map-positive sheep faeces and using PLS-DA score plots as was performed for the cattle faecal analysis. The PLS-DA plot of the sheep faeces (Figure 5A) showed a clear separation between Map-positive and Map-negative sheep faeces. Out of the 72 detectable VOCs, the loading plot for the sheep faeces (Figure 5B,C) revealed eight putative biomarkers that could be used to differentiate between Map-positive and Map-negative sheep faeces (Table 2). These putative biomarkers are UK2 (methanthiol), UK6 (dimethyl sulfide), UK7 (ethanthiol), UK26 (disulfide dimethyl), UK30 (1-butanol 3-methyl), UK57 (dimethyl trisulfide), UK70 (phenol), and UK72 (phenol 3-methyl). Five putative biomarkers, UK2, 26, 57, 70, and 30, were upregulated in the sheep positive samples, and three putative biomarkers UK6, 7, and 72, were upregulated in the negative samples.

Based on the calculations, the predicted sensitivity of the test is 100% and the predicted specificity is 100%. The predicted class error for the model is 0%. There were four compounds present in the headspace of both cattle and sheep faeces: methanthiol (UK2), dimethyl sulfide (UK6), phenol-3-methyl (UK72), and ethanthiol (UK7). There were six compounds present in the cattle faeces that were not present in the sheep faeces: acetone (UK5), 2-butanone (UK15), 2-butanol (UK16), 3-carene (UK49), p-cymene (UK64), and D-limonene (UK65); and there were four compounds present in the sheep faeces that were not present in the cattle faeces: disulfide dimethyl (UK26), dimethyl trisulfide (UK57), phenol (UK70), and 1-butanol 3-methyl (UK30). There was only one compound, 1-butanol 3-methyl (UK30), that was present in both sheep culture samples and sheep faecal samples.

### 3.5. Assessment and Validation of the eNose

**Figure 6:**
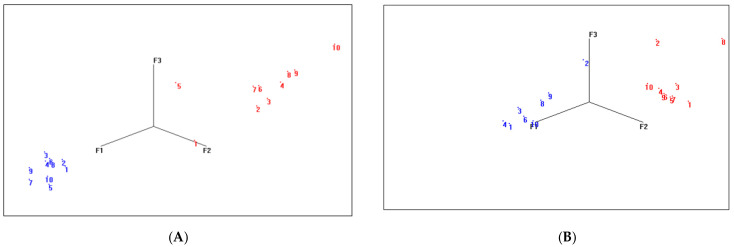
(**A**) Two-dimensional principal component analysis (PCA) scores plot of the Map-infected and non-infected cattle faeces used in the training set as determined by the eNose software. Red represents positive Map faecal samples 1–10 and blue represents negative Map faecal samples 1–10. (**B**) Two-dimensional principal component analysis (PCA) scores plot of the Map-infected and non-infected sheep faeces used in the training set as determined by the eNose software. The software displays three principal components (factors), i.e., F1, F2, and F3.

The eNose was first trained with 10 Map-positive and 10 Map-negative cattle faeces in the laboratory to determine whether the eNose could discriminate between infected and non-infected cattle. PCA of the raw data from cattle faeces obtained with the eNose showed clear separation between infected (red) and non-infected (blue) classes (Figure 6A). Based on PCA, a discrimination model was built. Internal cross-validation of the model then resulted in 100% successful discrimination between the two classes, with an interclass M-distance of 7.822 (M-distance ≥5 indicates good discrimination between classes, according to manufacturer’s instructions). The eNose was also trained with 10 Map-positive and 10 Map-negative sheep faeces in the laboratory. PCA of the data from sheep faeces obtained with the eNose showed clear separation between infection (red) and non-infected (blue) classes (Figure 6B). Internal cross-validation of this model also resulted in 100% successful discrimination between the two classes, with an interclass M-distance of 6.149.

**Figure 7:**
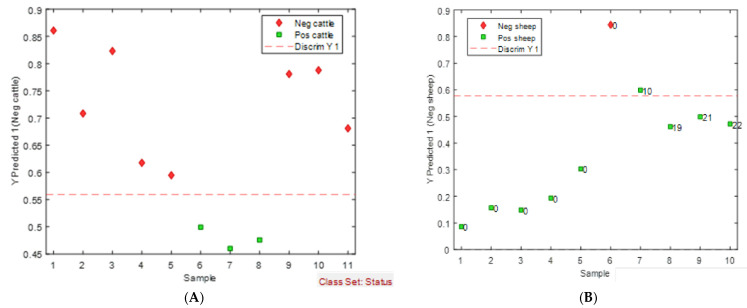
(**A**) The PLS-DA predicted model plot showing test sample prediction, Map-positive (green square) and Map-negative (red diamond) cattle faeces (eNose data analysed using Matlab and PLSToolbox). (**B**) The PLS-DA predicted model plot showing test sample prediction, Map-positive (green square) and Map-negative (red diamond) sheep faeces (eNose data analysed using Matlab and PLSToolbox).

Further validation of the eNose was conducted in the laboratory to ensure robustness and confidence in the ability of the eNose to identify Map-positive and Map-negative samples. For cattle faeces, the mean-centred data produced the best model (Figure 7A). This PLS-DA model used cross validation with venetian blinds (with 10 splits and a blind thickness of 1). The classification errors for the calibration, cross-validation, and prediction were 14.5%, 22.1%, and 0.0%, respectively. The predicted sensitivity of this test model is 100%, and the predicted specificity is 100%. Permutation testing (*n* = 50) returned *p*-values less than 0.05 (Wilcoxon test), suggesting that the model was not overfitted.

For the sheep faeces, the best model obtained was with mean-centred pre-processing and the PLS-DA plot (Figure 7B) showed good separation between positive and negative sheep faeces, resulting in the eNose having a predicted sensitivity of 100%, a specificity of 88.9%, and a class error of 5.5%.

## 4. Discussion

This study represents the first report of using VOC profiles for the detection of Map in both cattle and sheep faeces. It is also the first time that a portable electronic nose has been trained for detection of Map in cattle and sheep.

Map infection causes chronic inflammation of the small intestine, the jejuna, ileal mucosa, and lymph nodes along the colon [25]. Once Map is transferred into the intestine, it will eventually be excreted in the animal’s faeces [26]. VOCs originating from faeces therefore are potentially a good target for detecting Map. These VOCs may originate from Map itself, from intestinal inflammation, result from a local immune response, or result from the intestinal content itself (e.g., digested food, other intestinal bacteria).

Currently the gold standard for detection of Map is culture, which has high specificity but a lower sensitivity due to the intermittent shedding of the organism, formation of clumps, and the decontamination process required for culture [27]. Diagnosis of JD is time-consuming; however, the culture of Map can take from two to eight months to grow [28], prolonging the time to diagnosis. More rapid techniques like direct faecal qPCR can detect dead cells of the pathogen and so may detect passive shedders (faecal ‘pass-through’ phenomenon) [26], giving rise to false-positive results. Detection of smell-prints does not have these drawbacks as they are analysing volatile organic compounds that are being emitted by either or both the pathogen or the infected animal at the time of sampling.

To determine if the culture technique could be used with VOC detection to predict Map-positive and negative cultures with reduced times, GC-MS analysis was performed on the headspace of cultures at two-week intervals, starting from 2 weeks and finishing at 12 weeks. Sheep and cattle samples were analysed separately so that variations specific to the host species could be detected. Through PCA and loading plots, seven putative biomarkers were identified in cattle cultures (pentanal (UK23), hexanal (UK40), 1-octan-3-ol (UK59), 6-octen-2-one (Z) (UK60), 2-octanone (UK61), 1-butanol 3-methyl (UK30), and 3-pentanone (UK24)). Four of the compounds were upregulated in the positive cattle cultures, and four were upregulated in the negative cattle cultures and these seven compounds could be used to discriminate between positive and negative cultures.

Eight putative biomarkers were identified in the sheep cultures (dimethylamine (UK3), pentanal (UK23), hexanal (UK40), 6-octen-2-one (Z) (UK60), 2-octanone (UK61), 1-butanol 3-methyl (UK30), 1-butanol 2-methyl (UK38), and 3-pentanone (24)). Six compounds were upregulated in the positive sheep cultures, and two were upregulated in the negative sheep cultures. These eight putative biomarkers could be used to differentiate between positive and negative sheep cultures. Three compounds (dimethylamine, 1-butanol 2-methyl, and 1-octen-3-ol) were only identified in either the Map-infected cattle or sheep samples and could potentially be used to differentiate between Type-S and Type-C Map strains. Similar compounds have been associated with Map cultures. For example, dimethyl disulfide, pentanal, 1-octen-3-ol, 2-octanone, 1-butanol 3-methyl, 1-butanol 2-methyl, 3-pentanone, and hexanal have been identified in Map cultures [8,29] and were proposed as potential putative biomarkers for detecting early growth of Map [30].

The time to detect Map from culture samples using VOC analysis was reduced by up to a half. The normal time for detection of Map by routine culture is 12 weeks [31]. For cattle cultures, VOC was able to discriminate between positive and negative Map at four weeks and in sheep at six weeks post-incubation.

Only faeces whose results were the same for both culture and qPCR were included in the VOC analysis to ensure there was no ambiguity between a true-positive and a true-negative test result. For diagnostic purposes, test accuracy and validity are crucial when developing a new test method. Therefore, PLS-DA was used as a statistical method that allows training of the model and then validation against other samples to determine if the model could be used for diagnostic purposes. PLS-DA is a supervised dimensionality reduction method that includes class labels (e.g., positive vs. negative) in the analysis [32]. PLS-DA identifies components that maximise the separation between the classes and can determine the putative biomarkers that can differentiate between positive and negative samples more effectively and predict the effectiveness of the test in a diagnostic situation. Four of the compounds that were identified in the cattle faeces, 3-carene, acetone, 2-butanol, and 2–butanone, have been identified previously in VOC analysis of Map in goats [33] and in cattle [34], suggesting that these compounds may result from similar metabolic and pathophysiologic processes. Mycobacteria have also been shown to produce and grow on a variety of methyl ketones, such as 2-butanone and acetone [35], and have been shown to have more rapid and abundant growth in culture medium with the addition of these ketones [36]. Acetone is also linked to fat catabolism in both cattle [37] and humans [38] and is therefore most likely present due to the host response. To date, there has been no literature on the identification of the VOCs ethanthiol, p-cymene, D-limonene or phenol 3-methyl in Map faeces that have been identified as putative biomarkers of Map infection in this study.

PLS-DA evaluation of the test method for identifying putative biomarkers to differentiate between Map-positive and Map-negative cattle faeces resulted in a high sensitivity and specificity of 92.3% and 98.1%, respectively. These results, along with the low class error of 4.8%, indicate that VOC analysis of headspace in cattle faeces has a higher discriminatory power than current diagnostic tests such as indirect ELISA that are known to have sensitivities ranging from 16% to 34.9% [39]. Culture is reported to be almost 100% specific but only has a sensitivity of 50% due to a number of factors, including intermittent shedding of Map, formation of clumps, and the decontamination process during culture preparation [27].

PLS-DA and loading plots identified eight putative biomarkers that could be used to discriminate between Map-positive and negative sheep faeces. As this is the first time that VOCs have been identified in sheep faeces, it is not clear what processes these compounds are involved in in Map infection in sheep. PLS-DA evaluation of the test method for identifying putative biomarkers to differentiate between Map-positive and Map-negative sheep faeces resulted in 100% sensitivity and specificity, with no class error. This suggests that this method has a very high discriminatory power for detection of Map in sheep faeces that may be better than all other current diagnostic tests, including the gold standard culture. However, the evaluation of the test method in sheep included only a limited number of positive sheep faeces available for testing, with a total of 34 sheep faeces that consisted of only 4 positive and 30 negative faeces. In Australia, the vaccination rate for sheep is very high; a study of vaccinations from 1999 to 2009 identified that 91% of sheep were vaccinated in high-prevalence Map areas in New South Wales [40]. Therefore, finding unvaccinated sheep for use in research trials is very difficult. However, the preliminary data presented in this study offer promising prospects for the use of this test, and once further studies have been conducted on a larger number of sheep faecal samples, this method could be used in a diagnostic laboratory. Due to the high discriminatory power of both the cattle and sheep methods, it would be highly recommended to perform longitudinal studies to identify if this diagnostic tool could detect Map in subclinical animals.

PLS-DA and loading plots identified four common putative biomarkers (dimethyl sulfide (UK6), ethanthiol (UK7), phenol-3-methyl (UK72), and methanethiol (UK2)) in both Map-positive cattle and sheep samples. These putative biomarkers may represent a VOC profile that could be used to identify Map in both animal species. Both methanethiol and dimethyl sulfide have been previously identified as bacterial metabolites and may be produced by Map or its concentration altered due to Map [41]. Several bacterial species can produce and degrade methanethiol [41] and convert it to sulphur compounds, including dimethyl sulfide [42]. Phenol-3-methyl, also known as m-cresol, is a metabolite of aromatic amino acids produced by gut bacteria [41], and its regulation may be a direct or indirect effect of map. There is no literature on the presence of ethanthiol in faeces; therefore, it is uncertain if the presence of this putative biomarker is due to Map, the animal host and its microbiome, or some other source.

Point-of-care testing for the rapid detection of diseases near the patient enables better disease diagnosis and management. This is particularly important with a disease like JD that is spread rapidly on a farm and has no treatment or cure. This is the first study that has reported the use of a point-of-care gas-sensing system (eNose) for the detection of Map in sheep and cattle faecal samples. PCA and canonical discriminate analysis were used to develop a model that could differentiate between Map-infected and non-infected cattle and sheep faecal samples. CDA indicated that there were enough VOCs present in the samples to allow for discrimination between Map-positive and Map-negative faeces. Further validation of the models demonstrated that the eNose can discriminate between Map-infected and non-infected sheep and cattle faeces. The specificity of the eNose to detect Map-infected faeces was high for both sheep and cattle, being 88.9% and 100%, respectively. In sheep and cattle, the sensitivity of the eNose was 100%. The eNose only allows for 20 samples to be used for training (10 negative and 10 positive), and therefore, with this limited number, the PCA may have overfitted the data; however, the PLS-DA plots show that good models can be obtained.

The specificity of the eNose to accurately identify Map in sheep faeces will require further refinement. One of the limitations of this study was the availability of fresh sheep faecal samples, as frozen samples do not retain enough sensitivity to be evaluated using the eNose. With further evaluation of the statistical models with a larger number of sheep faeces and further development of the pre-processing and methodology, improvements in the specificity may be achieved. Point of care diagnosis using the Cyranose^®^ 320 eNose^®^ in the field will be a valuable option for diagnosis of JD on farm to allow for quick management decisions and to show instant proof of freedom of JD for farms participating in JD control programmes. This method of detection is also easy to perform and does not require specialist technical skills; it is cost-effective and less time-consuming, and it could potentially negate the need for culture, which is expensive and takes months for a result.

## 5. Conclusions

In this study, the detection of specific VOCs in the headspace of both cattle and sheep cultures resulted in the identification of putative biomarkers that could distinguish between Map-positive and Map-negative cultures. The time to detection of Map in cultures was reduced from 12 weeks to 4 weeks in cattle cultures and to 6 weeks in sheep cultures. Detection of specific VOCs in the headspace of cattle faeces has also been shown to be highly sensitive and specific for differentiating Map-infected from non-infected cattle faeces with a low error rate. There were several compounds identified that have been previously identified in Map infection in cattle and goats [32,33,34], showing a consistency in the VOC emissions from Map-infected cattle and sheep. Eight specific VOCs were identified for potential use as putative biomarkers for the detection of Map in sheep faeces, and the sensitivity and specificity of the test were 100%, indicating that this method has the potential to be used as a diagnostic tool once further evaluation has been performed on a larger data set. There were four common putative biomarkers identified in both cattle and sheep faeces that could be used as biomarkers for the detection of Map in both animal species. Analysis of the eNose data resulted in a specificity of 88.9% and a sensitivity of 100% in sheep, and in cattle, a specificity and sensitivity of 100%. This is the first time that a JD diagnostic test has been shown to be 100% in both sensitivity and specificity, and with such high sensitivity in both the sheep and cattle models, both the eNose and the GC-MS methods have a high potential to detect Map in subclinical animals. VOC emission patterns can also provide valuable information on alterations of metabolic pathways, which could potentially be used for vaccine development and for more accurate detection of Map in subclinically diseased animals.

## 6. Patents

This work is protected by an Australian provisional patent entitled “Methods of detecting *Mycobacterium avium* subsp. *paratuberculosis*” (application number: AU 2024903154).

## Figures and Tables

**Table 1:** Flow and data processing settings used for the Cyranose^®^ 320 eNose^®^ for optimal differentiation between Map-positive and Map-negative classes of cattle and sheep faeces.

Flow Settings		
	Time (s)	Pump Speed
Baseline purge	40	Medium
Sample draw	30	Medium
Sample draw 2	0	Medium
Snout removal	0	
1st sample gas purge	45	High
1st air intake purge	10	High
2nd sample gas purge	45	High
2nd air intake purge	0	High
Digital filtering	On	
Substrate heater	On 35C	
Training repeat count	1	
Identifying repeat count	1	
Data Processing		
Active sensors	All	
Algorithm	Canonical	
Pre-processing	Auto-scaling	
Normalisation	Normalisation 1	
Identification quality	Medium	
Acceptance threshold	99.900%	

**Table 2:** Significant compounds identified in cattle and sheep, their base peak ions (*m*/*z*), retention times (RT), Match, RMatch, and probability as identified in the NIST spectral library, identified from cattle culture (CC), sheep culture (SC), cattle faeces (CF), and sheep faeces (SF), and identification status based on library match (LM), confirmed: matches spectrum and RT of authentic standard. Refer to loading plots for directionality of regulation (i.e., up in positive or up in negative). Refer to Supplementary Figure S1 for chromatographic peak spectra of each putative biomarker.

ID No UK#	Name	*m/z*	RT (min)	Match	RMatch	Probability	Identified from	Identification Status
2	methanethiol	48.0014	3.949	510	765	68.3	SF, CF	LM
3	dimethylamine	45.0333	5.387	987	996	45.8	SC	LM
5	acetone	58.0414	6.223	728	948	51.9	CF	Confirmed
6	dimethyl sulfide	62.0176	6.315	896	901	71.6	CF, SF	LM
7	ethanthiol	62.0167	6.322	810	810	22.2	CF, SF	Confirmed
15	2-butanone	72.0570	10.771	699	879	56.7	CF	LM
16	2-butanol	45.0334	11.389	851	936	69.6	CF	Confirmed
23	pentanal	58.0411	16.489	874	881	40.7	CC, SC	Confirmed
24	3-pentanone	57.0322	16.673	855	912	67.8	SC, CC	LM
26	disulfide dimethyl	93.9902	19.015	868	868	93.3	SF	LM
30	1-butanol 3-methyl	55.0539	19.953	896	900	39.9	SC, CC, SF	Confirmed
38	1-butanol 2-methyl	55.0540	21.988	870	927	48.4	SC	Confirmed
40	Hexanal	56.0617	23.190	932	945	94.4	CC, SC	Confirmed
49	3-carene	93.0679	28.772	882	886	9.17	CF	Confirmed
57	dimethyl trisulfide	125.9597	30.746	762	867	94.4	SF	LM
59	1-octen-3-ol	57.0322	30.967	754	836	64.4	CC	Confirmed
60	6-octen-2-one (Z)	67.0528	31.053	780	805	54.8	SC, CC	LM
61	2-octanone	58.0400	31.094	858	858	71.8	SC, CC	Confirmed
64	p-cymene	119.0852	31.325	889	933	39.1	CF	Confirmed
65	D-limonene	93.0695	31.352	719	865	10.1	CF	Confirmed
70	phenol	94.0393	31.989	920	932	69.6	SF	Confirmed
72	phenol 3-methyl	107.0468	32.9	793	885	34.5	CF, SF	LM

## Data Availability

There are no new data associated with this article.

## Supplementary Materials

The following supporting information can be downloaded at: https://www.mdpi.com/article/10.3390/s24237443/s1. Supplementary Table S1: Map isolates used for the development and validation of the GC-MS analysis and eNose, including the PCR and culture results. Supplementary Table S2: IS900 PCR results for cultures at each time point: W2, W4, W6, W8, 10, and W12. Supplementary Figure S1: Chromatographic peak spectra of each putative biomarker described in Table 2.
