# Supplementary material for: Detection of Mycobacterium avium subsp. paratuberculosis in Australian Cattle and Sheep by Analysing Volatile Organic Compounds in Faeces
Source: Sensors (Basel). 2024 Nov 21;24(23):7443. doi: 10.3390/s24237443 (PMC11644260; doi:10.3390/s24237443)
Supplement: Supplementary file 1 [file sensors-24-07443-s001.zip › sensors-3263594-supplementary/sensors-3263594-supplementary_1.docx]

**Supplementary Table S1:** Map isolates used for the development and validation of the GC-MS analysis and eNose including the PCR and culture results

| **Isolate** | **Host** | **Year** | **Strain Type** | **Map Specific qPCR Result** | **Culture Result** |
| --- | --- | --- | --- | --- | --- |
| 51^1,2^ | Cattle | 2022 | C | Positive | Positive |
| 49^1,2^ | Cattle | 2022 | C | Positive | Positive |
| 34^1,2^ | Cattle | 2022 | C | Positive | Positive |
| 22^1,2^ | Cattle | 2022 | C | Positive | Positive |
| 18^1,2^ | Cattle | 2022 | C | Positive | Positive |
| 43^1,2^ | Cattle | 2022 | C | Positive | Positive |
| 10^1,2^ | Cattle | 2022 | C | Positive | Positive |
| 1^1,2^ | Cattle | 2022 | C | Positive | Positive |
| CNeg^1,2^ | Cattle | 2024 | C | Negative | Negative |
| S25^1,2,4^ | Sheep | 2023 | S | Positive | Positive |
| S26^1,2,4^ | Sheep | 2023 | S | Positive | Positive |
| SNeg^1,2^ | Sheep | 2024 | S | Negative | Negative |
| 3322-1^1,3,5^ | Sheep | 2023 | S | Negative | Negative |
| 3322-2^1,3,5^ | Sheep | 2023 | S | Negative | Negative |
| 3322-3^1,3,5^ | Sheep | 2023 | S | Negative | Negative |
| 3322-4^1,3,5^ | Sheep | 2023 | S | Negative | Negative |
| 3322-5^1,3,5^ | Sheep | 2023 | S | Negative | Negative |
| 3322-6^1,3,5^ | Sheep | 2023 | S | Negative | Negative |
| 3322-7^1,3,5^ | Sheep | 2023 | S | Negative | Negative |
| 3322-8^1,3,5^ | Sheep | 2023 | S | Negative | Negative |
| 3322-9^1,3.5^ | Sheep | 2023 | S | Negative | Negative |
| 3322-10^1,3,5^ | Sheep | 2023 | S | Negative | Negative |
| 232-3^1,4^ | Sheep | 2023 | S | Positive | Positive |
| 2534-20^1,4^ | Sheep | 2023 | S | Positive | Positive |
| 3833^1^ | Sheep | 2023 | S | Negative | Negative |
| 760-1^3,4,5^ | Cattle | 2024 | C | Positive | Positive |
| 760-2^3,4,5^ | Cattle | 2024 | C | Positive | Positive |
| 760-3^3,4,5^ | Cattle | 2024 | C | Positive | Positive |
| 760-4^3,4,5^ | Cattle | 2024 | C | Positive | Positive |
| 760-5^3,4,5^ | Cattle | 2024 | C | Positive | Positive |
| 760-7^3,4,5^ | Cattle | 2024 | C | Positive | Positive |
| 760-10^3,4,5^ | Cattle | 2024 | C | Positive | Positive |
| 760-11^3,4,5^ | Cattle | 2024 | C | Positive | Positive |
| 760-13^3,4,5^ | Cattle | 2024 | C | Positive | Positive |
| 760-16^3,4,5^ | Cattle | 2024 | C | Positive | Positive |
| 487-1^3,5^ | Cattle | 2024 | C | Negative | Negative |
| 487-2^3,5^ | Cattle | 2024 | C | Negative | Negative |
| 487-3^3,5^ | Cattle | 2024 | C | Negative | Negative |
| 487-4^3,5^ | Cattle | 2024 | C | Negative | Negative |
| 487-5^3,5^ | Cattle | 2024 | C | Negative | Negative |
| 487-6^3,5^ | Cattle | 2024 | C | Negative | Negative |
| 487-7^3,5^ | Cattle | 2024 | C | Negative | Negative |
| **Isolate** | **Host** | **Year** | **Strain Type** | **Map Specific qPCR Result** | **Culture Result** |
| 487-8^3^ | Cattle | 2024 | C | Negative | Negative |
| 487-9^3,5^ | Cattle | 2024 | C | Negative | Negative |
| 487-10^3,5^ | Cattle | 2024 | C | Negative | Negative |
| SA1^3,5^ | Sheep | 2024 | S | Positive | Positive |
| SA2^3,5^ | Sheep | 2024 | S | Positive | Positive |
| SA3^3,5^ | Sheep | 2024 | S | Positive | Positive |
| SA4^3,5^ | Sheep | 2024 | S | Positive | Positive |
| SA5^3,5^ | Sheep | 2024 | S | Positive | Positive |
| SA6^3,5^ | Sheep | 2024 | S | Positive | Positive |
| SA7^3,5^ | Sheep | 2024 | S | Positive | Positive |
| SA8^3,5^ | Sheep | 2024 | S | Positive | Positive |
| SA9^3,5^ | Sheep | 2024 | S | Positive | Positive |
| SA10^3,5^ | Sheep | 2024 | S | Positive | Positive |
| SA11^5^ | Sheep | 2024 | S | Positive | Positive |
| SA12^5^ | Sheep | 2024 | S | Positive | Positive |
| SA13^5^ | Sheep | 2024 | S | Positive | Positive |
| SA14^5^ | Sheep | 2024 | S | Positive | Positive |
| SA15^5^ | Sheep | 2024 | S | Positive | Positive |
| SA16^5^ | Sheep | 2024 | S | Positive | Positive |
| SA17^5^ | Sheep | 2024 | S | Positive | Positive |
| SA18^5^ | Sheep | 2024 | S | Positive | Positive |
| 760-8^4,5^ | Cattle | 2024 | C | Positive | Positive |
| 760-9^4,5^ | Cattle | 2024 | C | Positive | Positive |
| 760-13^4,5^ | Cattle | 2024 | C | Positive | Positive |
| 760-17^4,5^ | Cattle | 2024 | C | Positive | Positive |
| 760-18^4,5^ | Cattle | 2024 | C | Positive | Positive |
| 760-19^4,5^ | Cattle | 2024 | C | Positive | Positive |
| 760-20^4,5^ | Cattle | 2024 | C | Positive | Positive |
| 760-21^4,5^ | Cattle | 2024 | C | Positive | Positive |
| 760-22^4,5^ | Cattle | 2024 | C | Positive | Positive |
| 760-23^4,5^ | Cattle | 2024 | C | Positive | Positive |
| 760-25^4,5^ | Cattle | 2024 | C | Positive | Positive |
| 760-26^4,5^ | Cattle | 2024 | C | Positive | Positive |
| 760-27^4^ | Cattle | 2024 | C | Positive | Positive |
| 760-29^4^ | Cattle | 2024 | C | Positive | Positive |
| 760-30^4^ | Cattle | 2024 | C | Positive | Positive |
| 760-31^4^ | Cattle | 2024 | C | Positive | Positive |
| 760-32^4^ | Cattle | 2024 | C | Positive | Positive |
| 760-33^4^ | Cattle | 2024 | C | Positive | Positive |
| 1486-1^4,5^ | Cattle | 2024 | C | Negative | Negative |
| 1486-2^4,5^ | Cattle | 2024 | C | Negative | Negative |
| 1486-3^4,5^ | Cattle | 2024 | C | Negative | Negative |
| 1486-4^4,5^ | Cattle | 2024 | C | Negative | Negative |
| 1486-5^4,5^ | Cattle | 2024 | C | Negative | Negative |
| **Isolate** | **Host** | **Year** | **Strain Type** | **Map Specific qPCR Result** | **Culture Result** |
| 1486-6^4,5^ | Cattle | 2024 | C | Negative | Negative |
| 1486-7^4,5^ | Cattle | 2024 | C | Negative | Negative |
| 1486-9^4,5^ | Cattle | 2024 | C | Negative | Negative |
| 1486-10^4,5^ | Cattle | 2024 | C | Negative | Negative |
| 1486-11^4,5^ | Cattle | 2024 | C | Negative | Negative |
| 1486-12^4,5^ | Cattle | 2024 | C | Negative | Negative |
| 1486-13^4,5^ | Cattle | 2024 | C | Negative | Negative |
| 1486-14^4,5^ | Cattle | 2024 | C | Negative | Negative |
| 1486-15^4,5^ | Cattle | 2024 | C | Negative | Negative |
| 1486-16^4,5^ | Cattle | 2024 | C | Negative | Negative |
| 1486-17^4,5^ | Cattle | 2024 | C | Negative | Negative |
| 1486-18^4,5^ | Cattle | 2024 | C | Negative | Negative |
| 1486-19^4,5^ | Cattle | 2024 | C | Negative | Negative |
| 1486-20^4,5^ | Cattle | 2024 | C | Negative | Negative |
| 1486-21^4,5^ | Cattle | 2024 | C | Negative | Negative |
| 1486-22^4,5^ | Cattle | 2024 | C | Negative | Negative |
| 1486-23^4,5^ | Cattle | 2024 | C | Negative | Negative |
| 1486-24^4^ | Cattle | 2024 | C | Negative | Negative |
| 1486-25^4^ | Cattle | 2024 | C | Negative | Negative |
| 1486-26^4^ | Cattle | 2024 | C | Negative | Negative |
| 1486-27^4^ | Cattle | 2024 | C | Negative | Negative |
| 1486-28^4^ | Cattle | 2024 | C | Negative | Negative |
| 1486-29^4^ | Cattle | 2024 | C | Negative | Negative |
| 1486-30^4^ | Cattle | 2024 | C | Negative | Negative |
| 1486-31^4^ | Cattle | 2024 | C | Negative | Negative |
| 1486-32^4^ | Cattle | 2024 | C | Negative | Negative |
| 1486-33^4^ | Cattle | 2024 | C | Negative | Negative |
| 1486-34^4^ | Cattle | 2024 | C | Negative | Negative |
| 1486-35^4^ | Cattle | 2024 | C | Negative | Negative |
| 1486-36^4^ | Cattle | 2024 | C | Negative | Negative |
| 1486-37^4^ | Cattle | 2024 | C | Negative | Negative |
| 1486-38^4^ | Cattle | 2024 | C | Negative | Negative |
| 1486-39^4^ | Cattle | 2024 | C | Negative | Negative |
| 1486-40^4^ | Cattle | 2024 | C | Negative | Negative |
| 1486-41^4^ | Cattle | 2024 | C | Negative | Negative |
| 1486-42^4^ | Cattle | 2024 | C | Negative | Negative |
| 1486-43^4^ | Cattle | 2024 | C | Negative | Negative |
| 1486-44^4^ | Cattle | 2024 | C | Negative | Negative |
| 1486-45^4^ | Cattle | 2024 | C | Negative | Negative |
| 1486-46^4^ | Cattle | 2024 | C | Negative | Negative |
| 1486-47^4^ | Cattle | 2024 | C | Negative | Negative |
| 1486-48^4^ | Cattle | 2024 | C | Negative | Negative |
| 1486-49^4^ | Cattle | 2024 | C | Negative | Negative |
| 3322-11^4,5^ | Sheep | 2023 | S | Negative | Negative |
| **Isolate** | **Host** | **Year** | **Strain Type** | **Map Specific qPCR Result** | **Culture Result** |
| 3322-12^4,5^ | Sheep | 2023 | S | Negative | Negative |
| 3322-13^4,5^ | Sheep | 2023 | S | Negative | Negative |
| 3322-14^4,5^ | Sheep | 2023 | S | Negative | Negative |
| 3322-15^4,5^ | Sheep | 2023 | S | Negative | Negative |
| 3322-16^4,5^ | Sheep | 2023 | S | Negative | Negative |
| 3322-17^4,5^ | Sheep | 2023 | S | Negative | Negative |
| 3322-18^4,5^ | Sheep | 2023 | S | Negative | Negative |
| 3322-19^4,5^ | Sheep | 2023 | S | Negative | Negative |
| 3322-20^4,5^ | Sheep | 2023 | S | Negative | Negative |
| 3322-21^4,5^ | Sheep | 2023 | S | Negative | Negative |
| 3322-22^4,5^ | Sheep | 2023 | S | Negative | Negative |
| 3322-23^4,5^ | Sheep | 2023 | S | Negative | Negative |
| 3322-24^4^ | Sheep | 2023 | S | Negative | Negative |
| 3322-25^4^ | Sheep | 2023 | S | Negative | Negative |
| 3322-26^4^ | Sheep | 2023 | S | Negative | Negative |
| 3322-27^4^ | Sheep | 2023 | S | Negative | Negative |
| 3322-28^4^ | Sheep | 2023 | S | Negative | Negative |
| 3322-29^4^ | Sheep | 2023 | S | Negative | Negative |
| 3322-30^4^ | Sheep | 2023 | S | Negative | Negative |
| 3322-31^4^ | Sheep | 2023 | S | Negative | Negative |
| 3322-32^4^ | Sheep | 2023 | S | Negative | Negative |
| 3322-33^4^ | Sheep | 2023 | S | Negative | Negative |
| 3322-34^4^ | Sheep | 2023 | S | Negative | Negative |
| 3322-35^4^ | Sheep | 2023 | S | Negative | Negative |
| 3322-36^4^ | Sheep | 2023 | S | Negative | Negative |
| 3322-37^4^ | Sheep | 2023 | S | Negative | Negative |
| 3322-38^4^ | Sheep | 2023 | S | Negative | Negative |
| 3322-39^4^ | Sheep | 2023 | S | Negative | Negative |
| 3322-40^4^ | Sheep | 2023 | S | Negative | Negative |

^1^Isolates used for GC-MS method development on direct faeces,^2^Isolates used for GC-MS method development on cultures, ^3^Isolates used for training of the Cyranose® 320 eNose®, ^4^Isolates used for validation of the GC-MS method ^5^Isolates used for the validation of the Cyranose® 320 eNose®

**Supplementary Table S2:** IS900 PCR results for cultures at each time point, W2, W4, W6, W8, W10, W12

| **Isolate** | **Strain Type** | **Week 2** | **Week 4** | **Week 6** | **Week 8** | **Week 10** | **Week 12** |
| --- | --- | --- | --- | --- | --- | --- | --- |
| S25 | S | - | - | - | - | - | + |
| S26 | S | - | - | - | - | - | + |
| SNeg | S | - | - | - | - | - | + |
| CNeg | C | - | - | - | - | - | + |
| 51 | C | - | - | - | - | - | + |
| 49 | C | - | - | - | + | + | + |
| 34 | C | - | - | - | - | - | + |
| 22 | C | - | - | - | - | - | + |
| 18 | C | - | - | - | + | + | + |
| 43 | C | - | - | - | - | + | + |
| 10 | C | - | - | - | - | - | + |
| 1 | C | - | - | - | - | - | + |
