# Supplementary material for: Detection of Mycobacterium avium subsp. paratuberculosis in Australian Cattle and Sheep by Analysing Volatile Organic Compounds in Faeces
Source: Sensors (Basel). 2024 Nov 21;24(23):7443. doi: 10.3390/s24237443 (PMC11644260; doi:10.3390/s24237443)
Supplement: Supplementary file 1 [file sensors-24-07443-s001.zip › sensors-3263594-supplementary_S1.pdf]

Supplementary Table S1: Map isolates used for the development and validation of the GC-MS analysis and eNose including the PCR and culture results

Isolate	Host	Year	Strain Type	Map Specific qPCR Result	Culture Result
51[1,2]	Cattle	2022	C	Positive	Positive
49[1,2]	Cattle	2022	C	Positive	Positive
34[1,2]	Cattle	2022	C	Positive	Positive
22[1,2]	Cattle	2022	C	Positive	Positive
18[1,2]	Cattle	2022	C	Positive	Positive
43[1,2]	Cattle	2022	C	Positive	Positive
10[1,2]	Cattle	2022	C	Positive	Positive
1[1,2]	Cattle	2022	C	Positive	Positive
CNeg[1,2]	Cattle	2024	C	Negative	Negative
S25[1,2,4]	Sheep	2023	S	Positive	Positive
S26[1,2,4]	Sheep	2023	S	Positive	Positive
SNeg[1,2]	Sheep	2024	S	Negative	Negative
3322-1[1,3,5]	Sheep	2023	S	Negative	Negative
3322-2[1,3,5]	Sheep	2023	S	Negative	Negative
3322-3[1,3,5]	Sheep	2023	S	Negative	Negative
3322-4[1,3,5]	Sheep	2023	S	Negative	Negative
3322-5[1,3,5]	Sheep	2023	S	Negative	Negative
3322-6[1,3,5]	Sheep	2023	S	Negative	Negative
3322-7[1,3,5]	Sheep	2023	S	Negative	Negative
3322-8[1,3,5]	Sheep	2023	S	Negative	Negative
3322-9[1,3.5]	Sheep	2023	S	Negative	Negative
3322-10[1,3,5]	Sheep	2023	S	Negative	Negative
232-3[1,4]	Sheep	2023	S	Positive	Positive
2534-20[1,4]	Sheep	2023	S	Positive	Positive
3833[1]	Sheep	2023	S	Negative	Negative
760-1[3,4,5]	Cattle	2024	C	Positive	Positive
760-2[3,4,5]	Cattle	2024	C	Positive	Positive
760-3[3,4,5]	Cattle	2024	C	Positive	Positive
760-4[3,4,5]	Cattle	2024	C	Positive	Positive
760-5[3,4,5]	Cattle	2024	C	Positive	Positive
760-7[3,4,5]	Cattle	2024	C	Positive	Positive
760-10[3,4,5]	Cattle	2024	C	Positive	Positive
760-11[3,4,5]	Cattle	2024	C	Positive	Positive
760-13[3,4,5]	Cattle	2024	C	Positive	Positive
760-16[3,4,5]	Cattle	2024	C	Positive	Positive
487-1[3,5]	Cattle	2024	C	Negative	Negative
487-2[3,5]	Cattle	2024	C	Negative	Negative
487-3[3,5]	Cattle	2024	C	Negative	Negative
487-4[3,5]	Cattle	2024	C	Negative	Negative
487-5[3,5]	Cattle	2024	C	Negative	Negative
487-6[3,5]	Cattle	2024	C	Negative	Negative
487-7[3,5]	Cattle	2024	C	Negative	Negative

Isolate	Host	Year	Strain Type	Map Specific qPCR Result	Culture Result
487-8[3]	Cattle	2024	C	Negative	Negative
487-9[3,5]	Cattle	2024	C	Negative	Negative
487-10[3,5]	Cattle	2024	C	Negative	Negative
SA1[3,5]	Sheep	2024	S	Positive	Positive
SA2[3,5]	Sheep	2024	S	Positive	Positive
SA3[3,5]	Sheep	2024	S	Positive	Positive
SA4[3,5]	Sheep	2024	S	Positive	Positive
SA5[3,5]	Sheep	2024	S	Positive	Positive
SA6[3,5]	Sheep	2024	S	Positive	Positive
SA7[3,5]	Sheep	2024	S	Positive	Positive
SA8[3,5]	Sheep	2024	S	Positive	Positive
SA9[3,5]	Sheep	2024	S	Positive	Positive
SA10[3,5]	Sheep	2024	S	Positive	Positive
SA11[5]	Sheep	2024	S	Positive	Positive
SA12[5]	Sheep	2024	S	Positive	Positive
SA13[5]	Sheep	2024	S	Positive	Positive
SA14[5]	Sheep	2024	S	Positive	Positive
SA15[5]	Sheep	2024	S	Positive	Positive
SA16[5]	Sheep	2024	S	Positive	Positive
SA17[5]	Sheep	2024	S	Positive	Positive
SA18[5]	Sheep	2024	S	Positive	Positive
760-8[4,5]	Cattle	2024	C	Positive	Positive
760-9[4,5]	Cattle	2024	C	Positive	Positive
760-13[4,5]	Cattle	2024	C	Positive	Positive
760-17[4,5]	Cattle	2024	C	Positive	Positive
760-18[4,5]	Cattle	2024	C	Positive	Positive
760-19[4,5]	Cattle	2024	C	Positive	Positive
760-20[4,5]	Cattle	2024	C	Positive	Positive
760-21[4,5]	Cattle	2024	C	Positive	Positive
760-22[4,5]	Cattle	2024	C	Positive	Positive
760-23[4,5]	Cattle	2024	C	Positive	Positive
760-25[4,5]	Cattle	2024	C	Positive	Positive
760-26[4,5]	Cattle	2024	C	Positive	Positive
760-27[4]	Cattle	2024	C	Positive	Positive
760-29[4]	Cattle	2024	C	Positive	Positive
760-30[4]	Cattle	2024	C	Positive	Positive
760-31[4]	Cattle	2024	C	Positive	Positive
760-32[4]	Cattle	2024	C	Positive	Positive
760-33[4]	Cattle	2024	C	Positive	Positive
1486-1[4,5]	Cattle	2024	C	Negative	Negative
1486-2[4,5]	Cattle	2024	C	Negative	Negative
1486-3[4,5]	Cattle	2024	C	Negative	Negative
1486-4[4,5]	Cattle	2024	C	Negative	Negative
1486-5[4,5]	Cattle	2024	C	Negative	Negative

Isolate	Host	Year	Strain Type	Map Specific qPCR Result	Culture Result
1486-6[4,5]	Cattle	2024	C	Negative	Negative
1486-7[4,5]	Cattle	2024	C	Negative	Negative
1486-9[4,5]	Cattle	2024	C	Negative	Negative
1486-10[4,5]	Cattle	2024	C	Negative	Negative
1486-11[4,5]	Cattle	2024	C	Negative	Negative
1486-12[4,5]	Cattle	2024	C	Negative	Negative
1486-13[4,5]	Cattle	2024	C	Negative	Negative
1486-14[4,5]	Cattle	2024	C	Negative	Negative
1486-15[4,5]	Cattle	2024	C	Negative	Negative
1486-16[4,5]	Cattle	2024	C	Negative	Negative
1486-17[4,5]	Cattle	2024	C	Negative	Negative
1486-18[4,5]	Cattle	2024	C	Negative	Negative
1486-19[4,5]	Cattle	2024	C	Negative	Negative
1486-20[4,5]	Cattle	2024	C	Negative	Negative
1486-21[4,5]	Cattle	2024	C	Negative	Negative
1486-22[4,5]	Cattle	2024	C	Negative	Negative
1486-23[4,5]	Cattle	2024	C	Negative	Negative
1486-24[4]	Cattle	2024	C	Negative	Negative
1486-25[4]	Cattle	2024	C	Negative	Negative
1486-26[4]	Cattle	2024	C	Negative	Negative
1486-27[4]	Cattle	2024	C	Negative	Negative
1486-28[4]	Cattle	2024	C	Negative	Negative
1486-29[4]	Cattle	2024	C	Negative	Negative
1486-30[4]	Cattle	2024	C	Negative	Negative
1486-31[4]	Cattle	2024	C	Negative	Negative
1486-32[4]	Cattle	2024	C	Negative	Negative
1486-33[4]	Cattle	2024	C	Negative	Negative
1486-34[4]	Cattle	2024	C	Negative	Negative
1486-35[4]	Cattle	2024	C	Negative	Negative
1486-36[4]	Cattle	2024	C	Negative	Negative
1486-37[4]	Cattle	2024	C	Negative	Negative
1486-38[4]	Cattle	2024	C	Negative	Negative
1486-39[4]	Cattle	2024	C	Negative	Negative
1486-40[4]	Cattle	2024	C	Negative	Negative
1486-41[4]	Cattle	2024	C	Negative	Negative
1486-42[4]	Cattle	2024	C	Negative	Negative
1486-43[4]	Cattle	2024	C	Negative	Negative
1486-44[4]	Cattle	2024	C	Negative	Negative
1486-45[4]	Cattle	2024	C	Negative	Negative
1486-46[4]	Cattle	2024	C	Negative	Negative
1486-47[4]	Cattle	2024	C	Negative	Negative
1486-48[4]	Cattle	2024	C	Negative	Negative
1486-49[4]	Cattle	2024	C	Negative	Negative
3322-11[4,5]	Sheep	2023	S	Negative	Negative

Isolate	Host	Year	Strain Type	Map Specific qPCR Result	Culture Result
3322-12[4,5]	Sheep	2023	S	Negative	Negative
3322-13[4,5]	Sheep	2023	S	Negative	Negative
3322-14[4,5]	Sheep	2023	S	Negative	Negative
3322-15[4,5]	Sheep	2023	S	Negative	Negative
3322-16[4,5]	Sheep	2023	S	Negative	Negative
3322-17[4,5]	Sheep	2023	S	Negative	Negative
3322-18[4,5]	Sheep	2023	S	Negative	Negative
3322-19[4,5]	Sheep	2023	S	Negative	Negative
3322-20[4,5]	Sheep	2023	S	Negative	Negative
3322-21[4,5]	Sheep	2023	S	Negative	Negative
3322-22[4,5]	Sheep	2023	S	Negative	Negative
3322-23[4,5]	Sheep	2023	S	Negative	Negative
3322-24[4]	Sheep	2023	S	Negative	Negative
3322-25[4]	Sheep	2023	S	Negative	Negative
3322-26[4]	Sheep	2023	S	Negative	Negative
3322-27[4]	Sheep	2023	S	Negative	Negative
3322-28[4]	Sheep	2023	S	Negative	Negative
3322-29[4]	Sheep	2023	S	Negative	Negative
3322-30[4]	Sheep	2023	S	Negative	Negative
3322-31[4]	Sheep	2023	S	Negative	Negative
3322-32[4]	Sheep	2023	S	Negative	Negative
3322-33[4]	Sheep	2023	S	Negative	Negative
3322-34[4]	Sheep	2023	S	Negative	Negative
3322-35[4]	Sheep	2023	S	Negative	Negative
3322-36[4]	Sheep	2023	S	Negative	Negative
3322-37[4]	Sheep	2023	S	Negative	Negative
3322-38[4]	Sheep	2023	S	Negative	Negative
3322-39[4]	Sheep	2023	S	Negative	Negative
3322-40[4]	Sheep	2023	S	Negative	Negative

[1]Isolates used for GC-MS method development on direct faeces,[2]Isolates used for GC-MS method development on cultures, [3]Isolates used for training of the Cyranose® 320 eNose®, [4]Isolates used for validation of the GC-MS method [5]Isolates used for the validation of the Cyranose® 320 eNose®

Supplementary Table S2: IS900 PCR results for cultures at each time point, W2, W4, W6, W8, W10, W12

Isolate	Strain Type	Week 2	Week 4	Week 6	Week 8	Week 10	Week 12
S25	S	-	-	-	-	-	+
S26	S	-	-	-	-	-	+
SNeg	S	-	-	-	-	-	+
CNeg	C	-	-	-	-	-	+
51	C	-	-	-	-	-	+
49	C	-	-	-	+	+	+
34	C	-	-	-	-	-	+
22	C	-	-	-	-	-	+
18	C	-	-	-	+	+	+
43	C	-	-	-	-	+	+
10	C	-	-	-	-	-	+
1	C	-	-	-	-	-	+
