# Supplementary material for: Detection of Mycobacterium avium subsp. paratuberculosis in Australian Cattle and Sheep by Analysing Volatile Organic Compounds in Faeces
Source: Sensors (Basel). 2024 Nov 21;24(23):7443. doi: 10.3390/s24237443 (PMC11644260; doi:10.3390/s24237443)
Supplement: Supplementary file 1 [file sensors-24-07443-s001.zip › sensors-3263594-supplementary_S2.pdf]

Supplementary Figure S1: Chromatographic peak spectra of each putative biomarker described in Table two.

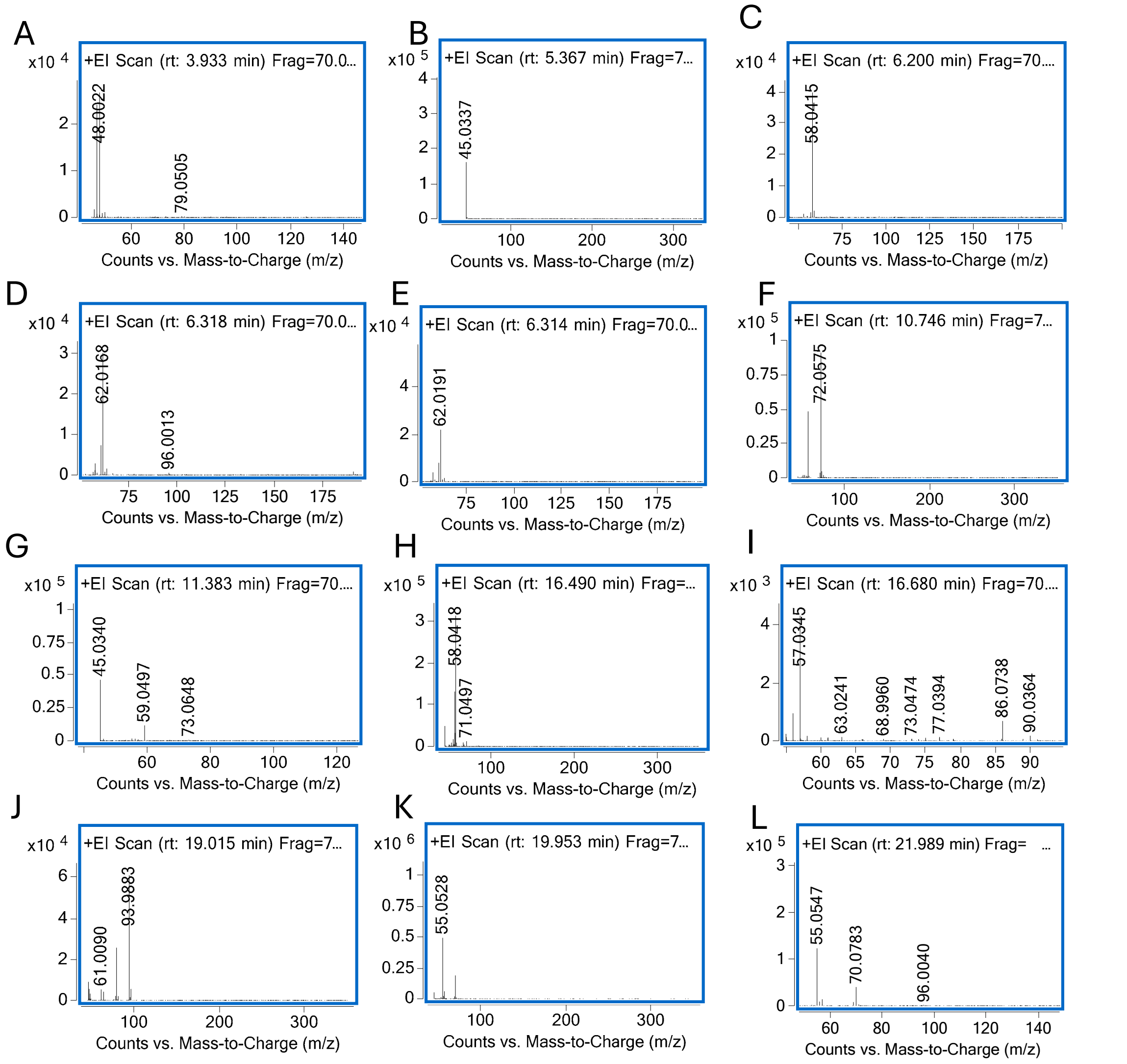

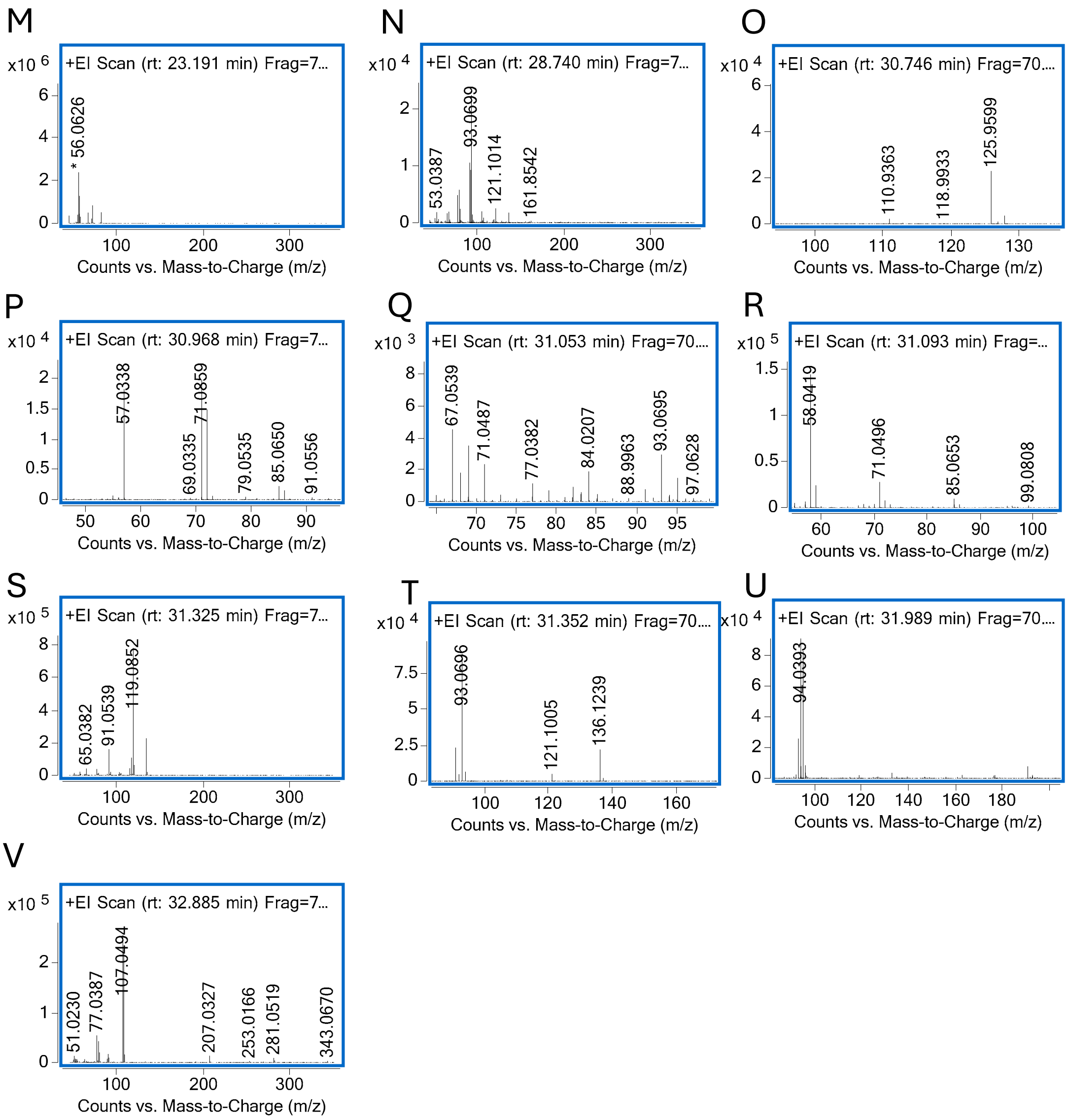

A = UK2, B = UK3, C = UK5, D = UK6, E = UK7, F = UK15, G = UK16, H = UK23, I = UK24, J = UK26, K = UK 30, L = UK 38, M = UK40, N = UK 49, O = UK57, P = UK59, Q = UK60, R = UK61, S = UK64, T = UK65, U = UK70, V = UK72
